# Predictors for the change in intimate partner violence among adolescent married girls aged 15–19 years: Estimates from random effect model

**DOI:** 10.1186/s12905-023-02252-z

**Published:** 2023-03-14

**Authors:** Debashree Sinha, Shobhit Srivastava, Muhammad T, Pradeep Kumar

**Affiliations:** 1 Department of Population & Development, International Institute for Population Sciences, Mumbai, 400088 Maharashtra India; 2 Department of Mathematical Demography & Statistics, International Institute for Population Sciences, Mumbai, 400088 Maharashtra India; 3 Department of Population Policies and Programs, International Institute for Population Sciences, Mumbai, 400088 Maharashtra India; 4 Specialist-Monitoring & Evaluation, Health Action Trust, Lucknow, Uttar Pradesh India

**Keywords:** Intimate Partner Violence, Married adolescent girls, Longitudinal study, UDAYA

## Abstract

**Background:**

Intimate Partner Violence (IPV) is one of the most common forms of violence against women. IPV against adolescents and young adult married women (15–19 years only) is poorly understood and not much researched as compared to their adult counterparts. The present study investigates the changes in multiple forms of IPV and tries to understand its association with different individual factors.

**Methods:**

The study used longitudinal data from Understanding the lives of Adolescent and Young Adults study (UDAYA), conducted in 2015-16 (wave 1) and 2018-19 (wave 2). The survey was done in two Indian states namely, Uttar Pradesh and Bihar. The sample size of the present study was 4,254 married adolescent girls aged 15–19 years. Multiple forms of IPV were the outcome variables of this study. A random effect regression analysis was used to estimate the association of changes in physical, sexual, and emotional violence with decision-making power and mobility restrictions along with other covariates.

**Results:**

Findings show that physical and emotional violence have increased from wave 1 to wave 2. Furthermore, married adolescent girls who took decisions alone/with others were less likely to suffer from IPV (β=-0.02; p < 0.05). Adolescent girls who agreed with the perception about wife-beating were more likely to report physical (β = 0.07; p < 0.05), sexual (β = 0.13; p < 0.05), and emotional violence (β = 0.14; p < 0.05). The risk of IPV was significantly more among adolescent girls whose family paid dowry compared to those who did not pay it (β = 0.04; p < 0.05).

**Conclusion:**

Interventions against those social norms that harm any female adolescents’ status in society and negatively impact their educational attainment should be adopted, simultaneously, with programs that promote gender equality in all aspects of their life.

## Introduction

According to the WHO (World Health Organization), Intimate Partner Violence (IPV) is one of the most common forms of violence against women. It includes physical and sexual violence, emotional and psychological abuse and controlling behavior by a current or former intimate partner [1]. Though the definition of an intimate partner varies across settings, it commonly refers to a partner in marriage, dating or cohabitation [2]. Globally, 30% of women aged 15 years and above [3] and over 35% in India [4] report having ever experienced any form of IPV. Furthermore, a multi-country study on gender-based violence against adolescent and young adult women in low- and middle-income countries confirms high prevalence of physical and sexual IPV [5]. It is also a serious public health concern. Studies show that IPV leads to death and disability [6, 7] and is associated with poor sexual and reproductive health, mental health, and self-rated health [8].

Several factors like age at marriage, education, socio-economic status, childhood experiences, perceptions about masculinity and belief in gender roles are found to be significant predictors of IPV. For instance, a study on Vietnamese adolescents found that early marriage, illiteracy, and exposure to sexual abuse to be associated with experience of IPV among young females [9]. A study on Indian young adult women of 15–24 years revealed young age at first marriage and parental IPV to be positively associated with all forms of IPV [10]. In yet another study on Indian women aged 20–24 years who were married as minors were found to be significantly more likely to report of ever experiencing marital violence than those married as adults [11]. Again, in Rajasthan, India women aged 20 to 24 years who married before the age of eighteen were more likely to have ever experienced IPV [12]. Likewise, a strong significant relationship between adolescent marriage and experience of physical IPV was found among young women of 20–24 years in Bangladesh [13].

Since, education provides a higher level of exposure to new gender norms [3], literary evidences across studies and settings show that high education act as a protective factor against IPV [14–17]. Similarly, a high socio-economic status is found to be a negative determinant of IPV [9, 18, 19]. Although, few studies indicate that with increase in spousal age difference IPV reduces [20, 21], in case of education gap, results are contradictory. For instance, few studies show that IPV increases if the woman is more educated than the man [20] while another study concludes the opposite i.e., wives with higher education than their husbands were less likely to experience less severe and severe IPV [22]. However, most of these studies are on adult women which creates a void in research focusing on adolescents and young women. Again, research on association of spousal age and education gap with IPV has received less attention and therefore, necessitates further investigation.

Empirical evidence suggests adolescents and younger women who are controlled by their husbands are more likely to face various forms of IPV [10, 23]. This can be either due to economic dependence of females over males within a family set up that contributes to perpetration and acceptance of IPV by men and women, respectively [24] or due to economic stress [25]. Moreover, working women (and therefore, economically independent) are more likely to face violence compared to their non-working counterparts [26, 27]. This unusual relationship is explained in terms of male backlash [28] which is deep rooted in the unequal power balance between males and females within the family and is widely observed in South Asian countries including India [29].

Equitable gender roles are known to be protective against IPV [30, 31] and belief in these gender roles are proposed as factors that favors tolerant attitudes towards violence against women [17, 32]. India is predominantly a patriarchal and patrilineal society with males being preferred and patronized since birth. Gender inequality in the power equations within the family shape attitudes and beliefs towards gender roles. A UNICEF report in 2012 highlights that more than 50% of adolescent males and females in India thought that wife beating was justifiable [33]. In fact, according to most of the adolescent students though sexual abuse or coercion was unacceptable, they were acceptable of physical and verbal abuse between ‘married couples’ [2].

Majority of the studies on socio-economic determinants of IPV in India focus on ever-married women in the reproductive age group [15, 19, 27, 28, 34]. Thus, IPV among adolescents and young adult married women (15–19 years only) are less understood and less researched compared to their adult counterpart. This gap in research on IPV have a bearing on how we control and manage our actions to prevent them, especially when every fifth person is an adolescent (10–19 years) in India. Furthermore, Uttar Pradesh (19.3%) and Bihar (9.2%) contributes to the highest total adolescent population in the country [35]. Additionally, research on IPV in patriarchal cultural settings like Uttar Pradesh and Bihar highlights the plight and vulnerability of women in context of incorrect reproductive health behavior, child mortality and other contextual factors [16, 18, 36–39]. Thus, an understanding of individual and household factors, attitudes and perception of adolescents towards IPV is essential in planning the control and prevention of this public health issue.

## Methods

### Data

Understanding the Lives of Adolescents and Young Adults (UDAYA), a longitudinal research on adolescents aged 10 to 19 years in Bihar and Uttar Pradesh, was used in the study. The study’s initial wave was performed in 2015-16, and a three-year follow-up survey was done in 2018-19. UDAYA provides estimates for the entire state as well as a sample of unmarried boys (10–14 years: younger boys and 15–19 years: older boys) and girls (10–14 years: younger girls and 15–19 years: older girls), as well as married females (15–19 years). The study used a multi-stage stratified sampling technique to draw sample regions for rural and urban areas separately. In each state, 150 primary sampling units (PSUs)—villages in rural regions and census wards in urban areas—were chosen as the sample frame, based on the 2011 census list of villages and wards. Households to be questioned were chosen through systematic sampling in each main sampling unit (PSU). More information on the research design and sample process may be found elsewhere [40]. A structured questionnaire was used to interview 20,594 adolescents in wave 1 (2015-16), with a response rate of 92%. Furthermore, in wave 2 (2018-19), the survey re-interviewed people who were successfully questioned in 2015-16 and who agreed to be interviewed again. The poll re-interviewed 4,567 boys and 12,251 girls out of a total of 20,594 who were eligible. After excluding the respondents who gave inconsistent response to age and education at the follow up survey (3%), the final follow-up sample covered 4,428 boys and 11,864 girls with the follow-up rate of 74% for boys and 81% for girls, respectively. The present study is based on married adolescent girls aged 15–19 years at wave 1 (N = 4,254).

A systematic review of IPV risk factors for the present study was undertaken. Through extensive review of literature and pertinent key word search, we tried to include all those risk factors of IPV in the analysis that was also available in the data set. A list of the variables that was included in the study is given below.

### Variable description

#### Outcome variables

1. Physical violence was categorized as 1 ‘Yes’ if the husband ever slapped, twisted or pulled hair, pushed/shook or threw something, kicked dragged beaten, burnt on purpose, attacked with a knife to the respondent and 0 ‘No,’ otherwise.
2. Sexual violence was defined as ‘Yes’ if the husband ever forced the respondent to have sex in the last 12 months and ‘No’; otherwise.
3. Emotional violence was defined as if the husband humiliate respondent in front of others coded as 1 ‘Yes’ and 0 ‘No,’ otherwise.
4. Intimate partner violence (IPV) was categorized as 1 “Yes” if the respondent experienced physical or sexual or emotional violence and 0 “no”, otherwise.

#### Explanatory variables

1. Decisions making power was categorized as “respondent alone/with others and other’s only”. The variable was generated using two questions (a) Who mainly takes the decision about making major household purchases? (b) Who mainly takes the decision about whether you should work or stay at home? The response were taken as “respondent only, jointly with others and others only”.
2. Mobility was categorized as “respondent alone/with others and other’s only”. The variable was generated using three questions (a) Are you usually allowed to go alone to a shop or market or visit a friend/relative inside your village/ward? (b) Are you usually allowed to a shop or market or visit a friend/relative outside your village/ward alone? (c) Are you usually allowed to attend any programme (a mela, sports event, girls’ group meetings) inside your village/ward alone? The responses were taken as “alone, only with someone else and not at all”.
3. Perception about wife beating was categorized as “respondent agree and disagree”. The variable was generated using the question “Is it all right for a husband to beat his wife if she doesn’t listen to him or obey him?”
4. Perception about money spend by husband alone was categorized as “no and yes”. The variable was generated using the question “Should husband alone/mainly decide how household money is to be spent?”
5. Paid work done by respondent was coded as “no and yes”.
6. Spousal age gap was categorized as “wife older or same age, Husband older by 1–2 years, Husband older by 3–4 years, Husband older by 5 + years”.
7. Spousal educational gap was categorized as “both not educated, wife more educated, husband more educated”.
8. Age at marriage was categorized as “less than 15 years and 15 years and more”.
9. Dowry during or after marriage was categorized as “no and yes”. The variable was generated using the question “Thinking about the time of your marriage, was a dowry paid to your husband/husband’s family at the time of your marriage or later?”
10. Wealth status was categorized as “poorest, poorer, middle, richer and richest”.
11. Religion was categorized as “Hindu and non-Hindu”.
12. Caste was categorized as “Scheduled Caste/Scheduled Tribe (SC/ST) and non-SC/ST”.
13. Place of residence as categorized as “urban and rural”.
14. States were provided as “Uttar Pradesh and Bihar”.

### Statistical analysis

At wave 1, descriptive analysis was used to look at the characteristics of married adolescent girls (2015-16). Changes in a few chosen variables were also detected from wave-1 (2015-16) to wave-2 (2018-19), and the significance of these changes was evaluated using the proportion test [41]. Moreover, random effect regression analysis [42, 43] was used estimate the association of changes in physical, sexual and emotional violence with decision making power and mobility restrictions among other covariates. Hausman test was performed to check whether fixed effect or random effect model was best fit model. The Hausman test [44, 45] revealed that random effect model was the best model for the analysis. Additionally, random effect model has certain benefit for the analysis of present paper that is its ability to estimate the effect of any variable that does not vary within clusters, which holds for all level 2 variables for e.g. dowry taken during or after marriage [46–48]. In the analysis we control for the variable dowry which is constant in wave-1 and wave-2 and hence random effect model will be the best model to estimate the effect of dowry on physical, sexual and emotional violence over married adolescent girls.

#### Ethical considerations

The ethical approval for this data (both wave 1 and wave 2) was provided by the Population Council Institutional Review Board. Adolescents provided individual written consent to participate in the study, along with a parent/guardian for adolescents younger than 18 years. The Population Council identified referral services for counselling and health services to offer respondents if necessary, and fieldworkers were trained on ethical issues and sensitivity. In addition, interviewing boys and girls in separate segments helped minimize issues related to confidentiality and response bias.

## Results

### Socio-economic profile of study participants, 2015-16 (*Table *1)

Among two per cent of participants, wife was older or same age as husband and in 22 per cent of cases, husband was older by 1–2 years than wife. In majority of cases husband was older than wife by five years (45 per cent). About 21 per cent of couples (both) had no education and among 32 per cent participants, wife was more educated than husband. Nearly 16 per cent adolescent girls were married before age 15 years. Moreover, dowry was paid by majority of the families of the study’s participant (86 per cent).

**Table 1:** Socio-economic profile of the study population, 2015-16

**Background characteristics**	**Adolescent married girls**	
	**Sample**	**Percentage**
**Spousal age gap**		
Wife older or same age	91	2.2
Husband older by 1–2 years	924	21.7
Husband older by 3–4 years	1331	31.3
Husband older by 5 + years	1908	44.9
**Spousal educational gap**		
Both not educated	911	21.4
Wife more educated	1346	31.6
Husband more educated	1997	47.0
**Age at marriage**		
Less than 15 years	696	16.4
15 or more years	3558	83.6
**Paid dowry**		
No	578	13.6
Yes	3676	86.4
**Wealth Index**		
Poorest	574	13.5
Poorer	884	20.8
Middle	974	22.9
Richer	1063	25.0
Richest	760	17.9
**Religion**		
Hindu	3,529	83.0
Non-Hindu	725	17.1
**Caste**		
SC/ST	1,242	29.2
Non-SC/ST	3,012	70.8
**Place of residence**		
Urban	556	13.1
Rural	3698	86.9
**States**		
Uttar Pradesh	2,774	65.2
Bihar	1,480	34.8
**Total**	4,254	100.0

### Summary statistics for the selected variables in Wave1 & Wave 2 (*Table *2)

Table 2 shows a significant increase in the decision making power of married adolescent girls (respondent alone/with others) between wave 1 & 2. Similar results was also observed for mobility (respondent alone/with others). The perception about wife beating for agree category increased from 73.4 per cent to 82.5 per cent married among adolescent girls between both survey waves. However, the perception about money spend by husband alone decreased by 4 per cent point. The percentage of married adolescent girls who were engaged in paid work increased from 11.2 per cent to 14.8 per cent between wave 1 & 2.

**Table 2:** Summary statistics for selected variables in wave-1 and wave-2

**Variables**	**Wave-1 (%)**	**Wave-2 (%)**
**Decision making power**		
Respondent alone/with others	61.5	65.0
Other’s only	38.5	35.0
**Mobility**		
Respondent alone/with others	95.7	98.9
Other’s only	4.3	1.1
**Perception about wife beating**		
Agree	73.4	82.5
Disagree	26.6	17.5
**Perception about money spend by husband alone**		
No	84.2	88.4
Yes	15.8	11.6
**Paid work by respondent**		
No	88.9	85.2
Yes	11.2	14.8
N (Total)	4254	4254

**Fig. 1:**
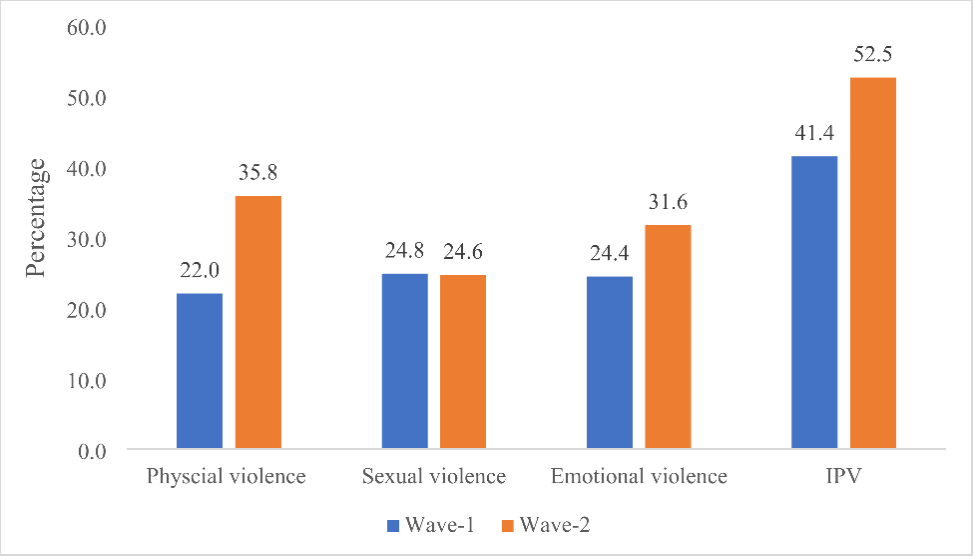
Percentage of married adolescent girls who experienced physical, sexual and emotional violence

Figure 1 displayed the prevalence of physical, sexual and emotional violence among married adolescent girls. The prevalence of physical violence among married adolescent girls was increased by about 14% point between wave 1 & 2. Similarly, the prevalence of emotional violence also increased from 24.4 per cent to 31.6 per cent during last three years. Increase in the prevalence of IPV was also in the same direction, i.e. a significant increase about 11% point between both waves.

Results from random effect estimates for physical, sexual, and emotional violence among married adolescent girls aged 15–19 years are presented in Table 3. The risk of physical (β = 0.14; p < 0.05), sexual (β = 0.03; p < 0.05), emotional (β = 0.10; p < 0.05) and intimate partner violence (IPV) (β = 0.13; p < 0.05) among married adolescent girls increased significantly during last three years (2015-16 to 2018-19). Married adolescent girls who took decision alone/with others were less likely to experience IPV compared to those whose decision was taken by other’s only (β=-0.02; p < 0.05). Similarly, adolescent girls who took decision about their mobility alone/with others were less likely to experience any kinds of violence. Adolescent girls who agreed with the perception about wife beating were more likely to report physical (β = 0.07; p < 0.05), sexual (β = 0.13; p < 0.05), emotional violence (β = 0.14; p < 0.05) and IPV (β = 0.14; p < 0.05) than those who disagreed that perception. The risk of all kinds of violence were more among married girls who engaged in paid work compared to those who were not engaged in paid work (each β = 0.05; p < 0.05). Adolescent girls whose husband were older by 3–4 years (β=-0.09; p < 0.05) or 5 + years (β=-0.10; p < 0.05) were less likely to report physical violence than those who were older or same age as their husband. Adolescent girls who married at age 15 years or more were less likely to report physical violence or IPV (each β=-0.09; p < 0.05) compared to those who were married before age 15 years. The risk of IPV was significantly more among adolescent girls whose family paid dowry compared to those who did not pay it (β = 0.04; p < 0.05). Adolescent girls who lived in rural areas were more likely to report all kinds of violence compared to those who lived in urban place of residence.

**Table 3:** Random effect estimates for physical, sexual and emotional violence among married adolescent girls aged 15–19 years

**Background characteristics**	**Physical violence**	**Sexual violence**	**Emotional violence**	**IPV**
	**Coef.**	**Coef.**	**Coef.**	**Coef.**
**Year**				
2015-16	Ref.	Ref.	Ref.	Ref.
2018-19	0.14*(0.12,0.16)	0.03*(0.01,0.05)	0.10*(0.08,0.11)	0.13*(0.11,0.15)
**Decision making power**				
Respondent alone/with others	Ref.	Ref.	Ref.	Ref.
Other’s only	-0.02(-0.04,0)	0.02*(0.01,0.04)	-0.01(-0.03,0.01)	-0.02*(-0.04,-0.01)
**Mobility**				
Respondent alone/with others	Ref.	Ref.	Ref.	Ref.
Other’s only	-0.08*(-0.15,-0.01)	-0.10*(-0.17,-0.03)	-0.06*(-0.13,-0.01)	-0.12*(-0.19,-0.04)
**Perception about wife beating**				
Disagree	Ref.	Ref.	Ref.	Ref.
Agree	0.07*(0.04,0.09)	0.13*(0.11,0.16)	0.14*(0.11,0.16)	0.14*(0.11,0.16)
**Perception about money spend by husband alone**				
No	Ref.	Ref.	Ref.	Ref.
Yes	-0.01(-0.04,0.02)	0.01(-0.02,0.04)	-0.04*(-0.07,-0.01)	-0.02*(-0.05,-0.01)
**Paid work by respondent**				
No	Ref.	Ref.	Ref.	Ref.
Yes	0.05*(0.02,0.08)	0.05*(0.02,0.08)	0.06*(0.03,0.09)	0.05*(0.02,0.08)
**Spousal age gap**				
Wife older or same age	Ref.	Ref.	Ref.	Ref.
Husband older by 1–2 years	-0.07(-0.16,0.01)	0.05(-0.03,0.14)	0.01(-0.08,0.09)	-0.02(-0.11,0.07)
Husband older by 3–4 years	-0.09*(-0.18,-0.01)	0.04(-0.04,0.12)	-0.01(-0.09,0.07)	-0.04(-0.12,0.05)
Husband older by 5 + years	-0.10*(-0.19,-0.01)	0.05(-0.03,0.13)	0.02(-0.08,0.08)	-0.04(-0.12,0.05)
**Spousal educational gap**				
Both not educated	Ref.	Ref.	Ref.	Ref.
Wife more educated	-0.03*(-0.05,-0.01)	-0.01(-0.04,0.02)	-0.03*(-0.06,-0.01)	-0.02(-0.05,0.01)
Husband more educated	-0.03*(-0.05,-0.01)	-0.02(-0.04,0.01)	-0.03*(-0.06,-0.01)	-0.02(-0.05,0.01)
**Age at marriage**				
Less than 15 years	Ref.	Ref.	Ref.	Ref.
15 or more years	-0.09*(-0.11,-0.06)	-0.01(-0.04,0.01)	-0.10*(-0.12,-0.07)	-0.09*(-0.11,-0.06)
**Paid dowry**				
No	Ref.	Ref.	Ref.	Ref.
Yes	0.02(-0.01,0.05)	0.03*(0.01,0.06)	0.02(-0.01,0.05)	0.04*(0.02,0.07)
**Wealth Index**				
Poorest	Ref.	Ref.	Ref.	Ref.
Poorer	-0.01(-0.05,0.02)	-0.01(-0.04,0.03)	0.01(-0.03,0.03)	-0.01(-0.04,0.03)
Middle	-0.04*(-0.07,-0.01)	-0.03*(-0.07,-0.01)	-0.03(-0.06,0.01)	-0.03(-0.06,0.01)
Richer	-0.08*(-0.11,-0.04)	-0.04*(-0.07,-0.01)	-0.05*(-0.08,-0.01)	-0.07*(-0.11,-0.03)
Richest	-0.14*(-0.19,-0.1)	-0.07*(-0.11,-0.03)	-0.11*(-0.15,-0.07)	-0.14*(-0.18,-0.1)
**Caste**				
SC/ST	Ref.	Ref.	Ref.	Ref.
Non-SC/ST	-0.05*(-0.07,-0.02)	-0.03*(-0.06,-0.01)	-0.03*(-0.05,0)	-0.03*(-0.05,0)
**Religion**				
Hindu	Ref.	Ref.	Ref.	Ref.
Non-Hindu	0.01(-0.03,0.03)	-0.04*(-0.07,-0.01)	-0.02(-0.05,0.01)	-0.02(-0.05,0.01)
**Place of residence**				
Urban	Ref.	Ref.	Ref.	Ref.
Rural	-0.05*(-0.08,-0.03)	-0.01(-0.04,0.01)	-0.03*(-0.05,-0.01)	-0.03*(-0.06,-0.01)
**States**				
Uttar Pradesh	Ref.	Ref.	Ref.	Ref.
Bihar	0.03*(0,0.05)	0.08*(0.06,0.1)	0.15*(0.12,0.17)	0.10*(0.07,0.12)
**Sigma_u**	0.181	0.112	0.106	0.139
**Sigma_e**	0.414	0.431	0.443	0.461
**Rho**	0.160	0.064	0.054	0.083
**Wald chi-square**	516.57*	335.78*	735.45*	628.24*
**Hausman test**	25.2	7.6	16.4	14.3

Coef: Coefficient; Ref: Reference: * if p > 0.05; IPV: Intimate partner violence; SC/ST: Scheduled Class/Scheduled Tribe.

## Discussion

Findings from this longitudinal study of IPV among married adolescent girls in two major states of India demonstrate an increased prevalence of IPV during the last three years between the survey rounds. Indicators of a woman’s autonomy/mobility and decision-making power were found to be negatively associated with past-year IPV in the present study which is similar in previous studies in India and other developing countries [49–51]. However, our finding is in variance with a study conducted in rural South India that found that violence against women increased with their greater involvement in income-generating activities either within or outside the home and control of the resultant income and perceptions of household economic responsibility [52].

Further, evidence in India suggests that gender-based violence is considered a male response to an increased modern attitude in women [53]. Research in India and poor resource countries also suggests that men sometimes use IPV to enforce their dominance and reassert unequal gender roles when women begin to become empowered through earning income [54, 55]. Consistently, the women participants in the present study who are engaged in the paid job were more likely to experience all types of physical, sexual, and emotional and an overall IPV. Similarly, husbands were oftentimes justified in beating their wives in circumstances such as her neglect of household duties, disobedience, or partners’ use of alcohol [56, 57]. In a cross-country study in Asia, an increasingly higher rate of approval for wife-beating was found in India with 57% of the women accepting it [58]. Consistent with past studies, respondents in our study who believed that wife-beating is not justified and disapproved of their husbands’ financial autonomy were more likely to experience all three types of violence and IPV [57]. The findings imply that as women begin to become empowered they start to question rigid gender roles but their empowerment may not be sufficient to protect them from the partner violence as a repercussion of their questioning. In this context, it is important to point out the difference when women versus men use violence – a battered women might also hit her partner e.g. in self-defence but the injuries and emotional and social impact will be less severe in a patriarchal society compared to when men use violence against women.

The results also indicate that marriage before the age of 15 among adolescent girls in India is highly prevalent similar to other resource-constrained settings [9, 59]. Young girls in Uttar Pradesh and Bihar in the present study who marry before the age of 15 were at increased risk of sexual, physical, and emotional violence and overall IPV experience in the last year. This can be related to the practice of early childhood marriage that leads to reduced educational opportunities for young girls compared to girls married during their older ages after settling their educational career; these girls may be less empowered and thus at increased risk of IPV after marriage [12]. The experience of violence and silent acceptance by women often undermine the attempts to bringing attitudinal changes and women empowerment and continue to be the barriers to the achievement of developmental goals [60]. Also, the pervasiveness of violence in communities with socially conservative attitudes towards adherence to longstanding gender norms, leads to its tolerance as normative behavior [14], confirming unconditional autonomy of men over their wives. Hence, the relative role of women in underplaying their empowerment and continuing to be subjected to IPV even when societal gender norms evolve has to be further investigated.

Furthermore, several studies have observed the effect of socioeconomic status on gender-based violence [17, 19, 61], indicating greater wealth and rural status as protective factors of IPV. Consistently, adolescent girls from better-off households had lower odds of partner violence in the present study. Education has also been one of the sources of women empowerment and has given them the ability to gather and assimilate information, and secure and protect themselves from multiple forms of violence [62–64]. Multiple studies observed that the prevalence of violence decreased along with the increase of women’s education and family income and a higher socioeconomic status was demonstrated as a protective buffer against domestic violence [15, 19, 65]. It is observed in our study that if husbands or wives were educated, respondents were less likely to experience sexual violence than if both were not educated. Furthermore, caste difference is found to play an important role with implications for educational and occupational stratification among women, which corresponds to increased violence against low-caste women [66]. Results have shown that being of the ethnicity of SC/ST, which are the most disadvantaged ethnic groups in India heightened the risk of IPV.

This study has considerable strengths. It included a large and systematically recruited nationally representative sample of adolescents, including the members of ethnic minorities. There were high recruitment and response rates in the survey data collected. Second, information about the experience of IPV and multiple types of violence was obtained by face-to-face interviews which proved to be a unique strength as well-trained interviewers not only explain IPV to the respondents that improves reporting, but also offer emotional support and signpost to services. However, we also acknowledge that information about the experience of IPV obtained in the face-to-face interview and not through self-reported questionnaires might have resulted in under-reporting among the respondents because of hesitance to disclose sensitive personal information and information about matters like sexual violence (which is generally considered to be shameful). Another limitation of the study is that the experiences of types of violence and IPV were only sought from ever-married adolescent participants, and the results, therefore, may not be generalizable to those in not unions as well as those in non-formal unions which are recently becoming common among adolescents in India. Also, the study does not acknowledge coercive control as a form of partner abuse. Future research on it would give a comprehensive picture on IPV against women in general and adolescents in particular.

## Conclusion

The findings highlight the importance of prevention efforts that aim to overcome gender-based multiple forms of partner violence. Interventions against the social norms that damage the female adolescents’ status in society and negatively impact their educational attainment should be adopted simultaneously with programs that promote gender equality in all aspects of adolescent’s life.

## Data Availability

Details of the data are provided in the methodology section. Data can be access through requests via the following URL: https://dataverse.harvard.edu/dataset.xhtml?persistentId=10.7910/DVN/RRXQNT.

## Abbreviations

IPV: Intimate Partner Violence
UDAYA: Understanding the lives of Adolescent and Young Adults study
SC/ST: Scheduled Caste/Scheduled Tribe
